# Acute liver failure due to primary angiosarcoma: A case report and review of literature

**DOI:** 10.1186/1477-7819-6-104

**Published:** 2008-09-30

**Authors:** Chandra S Bhati, Anand N Bhatt, Graham Starkey, Stefan G Hubscher, Simon R Bramhall

**Affiliations:** 1 Liver Unit, Queen Elizabeth Hospital, Birmingham, UK

## Abstract

**Background:**

Hepatic angiosarcoma is a primary sarcoma of the liver, accounting for only 2% of all primary hepatic malignancies. Acute liver failure is an extremely rare presentation of a primary liver tumour.

**Case presentation:**

We report a case of a seventy year-old man who presented with a very short period of jaundice leading to fulminant hepatic failure (FHF). On further investigation he was found to have primary angiosarcoma of liver.

**Conclusion:**

The treatment outcomes for hepatic angiosarcoma are poor, we discuss the options available and the need for prompt investigation and establishment of a diagnosis

## Background

Hepatic malignancies include primary hepatocellular carcinoma, metastases and primary or metastatic sarcomas [1]. Hepatic angiosarcoma is a primary sarcoma of the liver which accounts for only 2% of all primary hepatic malignancies [2-5]. Angiosarcoma is associated with environmental or occupational exposure to carcinogens (thorium dioxide, vinyl chloride, arsenic and radiation). There is also an association with hemochromatosis and von Recklinghausen disease [1,2,4]. In most cases of primary hepatic angiosarcoma, no obvious risk factor can be identified.

The most common causes of fulminant hepatic failure (FHF) are drug toxicity and sero-negative hepatitis [6]; rarer causes include Bud-Chiari syndrome and acute Wilson's disease. FHF can also develop very rarely as a consequence of primary or metastatic liver tumour, this generally occurs as a result of massive neoplastic infiltration of the hepatic sinusoids leading to secondary necrosis of hepatocytes [7]. Rowbotham et al reported 4020 cases of FHF, malignant infiltration accounted for only 0.44% (18 cases) [8].

There have been a number of case series reporting FHF secondary to infiltration of the liver by malignant cells [7-15], haematological malignancies are the most common [7-10]. Other infiltrative metastatic malignancies that rarely cause FHF include adenocarcinoma, melanoma, and anaplastic tumours [11-15]. Although hepatic dysfunction due to malignancy such as hepatocellular carcinoma or metastatic infiltration is common, acute liver failure in these cases is rare. We report a case of primary angiosarcoma of the liver which presented with FHF.

## Case presentation

A seventy year old Caucasian male, who had no significant previous medical history, was admitted to a local hospital with a history of sudden onset jaundice and weight loss. There was no previous history of jaundice or hepatitis. There was no significant history of alcohol in-take or exposure to arsenic, vinyl chloride, or Thorotrast. He never used any hepatotoxic or herbal medications and his mother died of undiagnosed liver disease.

Upon examination the patient was jaundiced without encephalopathy or focal neurological findings. He had bilateral pedal oedema and hepatomegaly. The patient did not have any other signs of liver failure. Liver function tests at admission revealed a total bilirubin of 203 mmol/dL (normal, 5–17 mmol/dL), aspartate aminotransferase (AST) 52 IU/L (normal, 4–44 IU/L), alkaline phosphatase 170 IU/L (normal, 67–213 IU/L), albumin 2.0 g/dL, PT 22 seconds, APTT 51 seconds and platelets 113,000/cm^3^.

**Figure 1:**
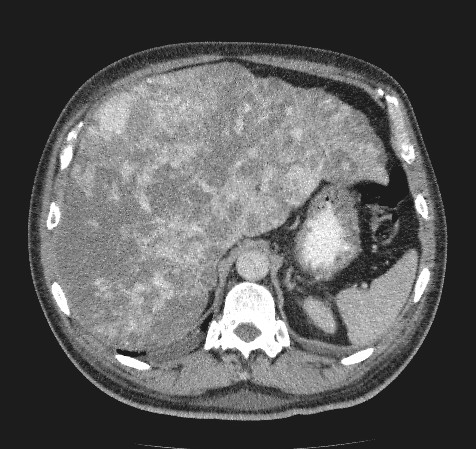
Abdominal CT scan showing complete replacement of liver parenchyma with liver tumour.

An urgent ultrasound scan demonstrated hepatomegaly with significant liver paranchymal alteration. A subsequent contrast enhanced abdominal CT showed gross replacement of liver with tumour tissue suggestive of a primary liver tumour (Figure 1). The patient was at this point referred to our centre.

The patient's initial evaluation in our Unit showed further derangement in the patients liver functions tests; total bilirubin had risen to 401 mmol/dL, AST to 132 IU/L, alkaline phosphatase to 370 IU/L and INR to 2.1. A local review of his CT scan raised the possibility of angiosarcoma. To confirm the diagnosis a transjugular biopsy was arranged as the clotting abnormality had been resistant to correction with fresh frozen plasma at the referring centre. Before this could be carried out patient rapidly deteriorated after admission and became progressively encephalopathic, consistent with FHF. He was treated conservatively with dextrose and broad spectrum antibiotics but deteriorated further and died two days after admission to the liver unit.

**Figure 2:**
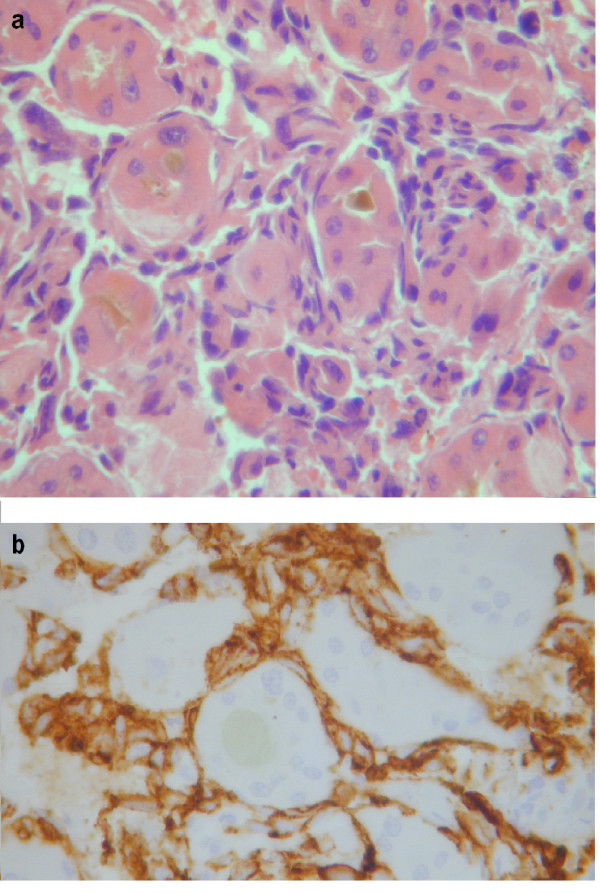
**(A) Liver biopsy showing sinusoidal infiltration by pleomorphic spindle cells typical of hepatic angiosarcoma.** There is disruption of the normal trabecular architecture with hepatocyes forming glandular structures containing bile plugs ("cholestatic rosettes"). **(B) **Spindle cells are strongly immunoreactive for the vascular endothelial marker CD34. (A = Haematoxylin and eosin, B = immunoperoxidase).

A post mortem liver biopsy was carried out confirming initial suspicions that this was a primary angiosarcoma of the liver. Microscopically, tumour was composed of poorly cohesive cells, oval to spindle shaped with high grade cytological atypia. The tumour had a sinusoidal growth pattern surrounding clusters of hepatocytes forming cholestatic rosettes (Figure 2a). Immunohistochemistry staining was strongly and diffusely positive for vascular endothelial markers (CD31, CD34) (Figure 2b) and for vimentin. Stains for the cytokeratins and hepatocyte specific antigen highlighted the presence of entrapped non neoplastic hepatocyte and bile ducts. Staining for smooth muscle actin appeared to be confined to areas of fibrotic tissue.

## Discussion

Angiosarcoma usually presents in late adulthood [2] with abdominal discomfort, distension, weight loss, and fatigue [4,16]. On examination, the patients may have jaundice, hepatomegaly, and ascites [4,16,17]. Our patient was admitted with similar symptoms. Fulminant hepatic failure (FHF) is defined as liver disease that results in encephalopathy within 28 days from the onset of jaundice in a patient with no prior evidence of liver disease. Presentation as FHF is rare, Table 1 shows published reports of clinical presentation and treatment of angiosarcoma in the current literature. In an adult FHF Study Group; acetaminophen overdose (46%), drug toxicity (11%) and hepatitis (10%) were found to be the most common causes for liver failure [18]. There are case reports where association of FHF with liver metastasis from other malignancies have been reported [7-15].

**Table 1:** Primary Angiosarcoma and fulminant liver failure and treatment

**Case series**	**No of patients**	**FHF**	**Treatment**	**Median Survival**
Monila et al [3]	5	No	1= R	6 mo
			2 = C	
			2 = N	
Forbes et al [16]	8	No	2 = OLTx	<30 days (OLTx) 1.7 mo (N)
			6 = N	
Poggio et al [17]	3	No	R	N/A
Rademaker et al [21]	4	No	N/A	N/A
Vennarecci et al [25]	6	No	4 = C	C = Max 8 mo
			2 = OLTx	Oltx = 10 mo
Husted et al [27]	6	N/A	OLTx	5.7 mo
Wiitz et al [28]	5	No	3 = R	11 months (R)
			2 = N	

R = Resection, N = No treatment, C = Chemotherapy, N/A = not available

The liver is commonly involved in metastatic disease, and the degree of liver biochemistry derangement tends to reflect the extent of parenchymal replacement with tumour [19]. In this patient, liver function tests were only slightly abnormal two weeks before development of FHF. Although, alteration of liver function tests in these patients is very common [20], liver failure is extremely rare.

CT scan is often diagnostic, demonstrating multiple hypodense areas typical of angiosarcoma. Post contrast, the lesions become partly or completely isodense compared with normal hepatic tissue [1,21]. In our patient liver parenchyma was completely replaced with tumour tissue (Figure 1).

The mechanism of liver failure is multifactorial. Evidence suggests a combination of hepatic ischaemia leading to parenchymal infarction, vascular occlusion of portal vein by tumour thrombi and nonocclusive infarction of liver due to shock from secondary causes such as sepsis or cardiac dysfunction plays an important role in these patients [12,22]. In this patient, replacement of hepatocytes by malignant cells, leading to secondary necrosis of hepatocytes played a significant role in development of liver failure.

Angiosarcoma has very limited treatment options, without treatment the majority of patients die within 6 months of diagnosis [4]. Surgery has a limited role due to the advanced stage at which these tumours present. Liver transplantation is contraindicated, as patients who have been transplanted incidentally have not shown any survival benefit. The data from European Liver Transplant Registry on 17 patients who had undergone transplantation for angiosarcoma had a median survival of only 7 months [23]. Hepatic resection has been reported in patients with limited disease but these results have also been poor. There are very few published case reports with good survival after liver resection (16 months [24] and 10 years [4]). The role of chemotherapy has been described with very limited improvement in overall length of survival [25]. Treatment with new techniques like transcatheter arterial chemoembolization (TACE) techniques has been described as a case report with very limited success in overall survival improvement [26].

## Conclusion

Our patient presented with mild hepatic failure that rapidly progressed to FHF. In the absence of a clear aetiology for FHF primary liver tumour must be considered in the differential diagnosis and a biopsy should be arranged to reach definitive diagnosis.

## Competing interests

The authors declare that they have no competing interests.

## Authors' contributions

CSB – Contributions to case selection, analysis and drafting the manuscript. ANB – Case analysis and initial drafting of manuscript. GS – Contributions to conception, arranging histopathology, revision of the manuscript. SGH – Histopathology evaluation, further study of slides and in depth analysis. SRB – Critical revision and final approval of the version to be published. All authors read and approved the final manuscript.

## Consent

Written informed consent was obtained from the patient for publication of this case report.
